# The N400 Effect during Speaker-Switch—Towards a Conversational Approach of Measuring Neural Correlates of Language

**DOI:** 10.3389/fpsyg.2016.01854

**Published:** 2016-11-28

**Authors:** Tatiana Goregliad Fjaellingsdal, Esther Ruigendijk, Stefan Scherbaum, Martin G. Bleichner

**Affiliations:** ^1^Department of Psychology, European Medical School, University of OldenburgOldenburg, Germany; ^2^Cluster of Excellence Hearing4all, University of OldenburgOldenburg, Germany; ^3^Department of Dutch, University of OldenburgOldenburg, Germany; ^4^Department of Psychology, Technische Universität DresdenDresden, Germany

**Keywords:** mobile EEG, prediction, N400, social interaction, conversation, language, dialogue, turn-taking

## Abstract

Language occurs naturally in conversations. However, the study of the neural underpinnings of language has mainly taken place in single individuals using controlled language material. The interactive elements of a conversation (e.g., turn-taking) are often not part of neurolinguistic setups. The prime reason is the difficulty to combine open unrestricted conversations with the requirements of neuroimaging. It is necessary to find a trade-off between the naturalness of a conversation and the restrictions imposed by neuroscientific methods to allow for ecologically more valid studies. Here, we make an attempt to study the effects of a conversational element, namely turn-taking, on linguistic neural correlates, specifically the N400 effect. We focus on the physiological aspect of turn-taking, the speaker-switch, and its effect on the detectability of the N400 effect. The N400 event-related potential reflects expectation violations in a semantic context; the N400 effect describes the difference of the N400 amplitude between semantically expected and unexpected items. Sentences with semantically congruent and incongruent final words were presented in two turn-taking modes: (1) reading aloud first part of the sentence and listening to speaker-switch for the final word, and (2) listening to first part of the sentence and speaker-switch for the final word. A significant N400 effect was found for both turn-taking modes, which was not influenced by the mode itself. However, the mode significantly affected the P200, which was increased for the reading aloud mode compared to the listening mode. Our results show that an N400 effect can be detected during a speaker-switch. Speech articulation (reading aloud) before the analyzed sentence fragment did also not impede the N400 effect detection for the final word. The speaker-switch, however, seems to influence earlier components of the electroencephalogram, related to processing of salient stimuli. We conclude that the N400 can effectively be used to study neural correlates of language in conversational approaches including speaker-switches.

## Introduction

The exchange of ideas via language is a central part of human culture. Understanding how these verbal interactions relate to the underlying brain processes is a challenge. This challenge arises from the open nature of conversations on the one hand, and on the restrictions imposed by neuroimaging techniques on the experimental setup on the other hand. Consequentially, to date, the neural underpinnings of natural conversations are poorly understood. To gain a better understanding it is therefore necessary to develop new experimental paradigms and new brain imaging setups.

Neuroscientific studies have taught us a considerable amount about the neurobiological basis of language (Friederici, 2011). Central in this research endeavor is the study of language processing in individuals using controlled language material. Natural language, however, is typically used during social interactions with one or more participants and we could gain more knowledge on language processing itself, if we find ways to study their neural underpinnings during conversations (Levinson, 2016).

Language development and language usage are fundamentally interactive: language is acquired interactively and is used in conversations with other people. These interactions are characterized by a back and forth between the conversational partners; we constantly switch between listening and speaking, we plan our next utterances and we build expectations, and even concrete predictions, about what our conversational partner will say. We give the other person time to speak and we anticipate those moments when it is our turn to speak (Levinson and Torreira, 2015; Torreira et al., 2015).

The large number of processes involved in a dialogue makes it very challenging to study conversations using neuroscientific measures. One challenge is the poor controllability of natural conversations. There is an evident tradeoff between the naturalness of a dialogue and possible experimental manipulations; any experimental manipulation leads almost inevitably to an unnatural dialogue. Another challenge are the restrictions and requirements that brain imaging techniques pose on the experimental setup (Van Berkum, 2012). All imaging techniques are sensitive to movement artifacts (e.g., speech related mouth movements) and therefore require the participant to move as little as possible, which also leads to an unnatural dialogue situation. Further, the small magnitude of the signal of interest generally requires many repetitions to increase the signal-to-noise ratio by data averaging (De Vos and Debener, 2014). What is needed are, first, paradigms that capture the aspects of natural conversations but retain sufficient structure to allow for quantitative analysis (e.g., Magyari et al., 2014; Mandel et al., 2015; Bögels et al., 2015a) and second, recording setups that allow for a more natural interaction, with less restrictions on the participants (e.g., Bašnáková et al., 2015; Bögels et al., 2015c). Our research aim is to move toward neurophysiological studies of natural dialogues. As a first step this is targeted, on the one hand, by incorporating a conversational element in the experimental paradigm and, on the other hand, by using an EEG device that enables a less restrained recording setup.

We present an experimental approach that entails a number of aspects that play a role in a conversation: speaking and listening, the formation of expectations during a conversation (i.e., how a sentence will end), and an element of turn-taking between speakers (Coates, 1990; Purver et al., 2009), that is, a speaker-switch, comparable to a situation in which one person completes the utterance of another person. In this paradigm participants either read out or listen to the pre-recorded first part of a sentence, the last word of the sentence is in both conditions presented by another speaker and either completes the sentence in an expected or an unexpected way, i.e., it matches or violates the semantic expectation of the participant. Finally, we use a wireless EEG setup that allows EEG recordings in less restricted ways, i.e., the amplifier is directly attached to the cap and does therefore not restrain the participant. In the long run, the application of wireless EEG enables to move the experiment outside the strongly controlled lab environment (e.g., De Vos et al., 2014). In this study, further aspects such as sequential full turns and other conversational elements (cf. Levinson and Torreira, 2015) are left aside.

The variable of interest in this paradigm is the N400 event-related potential (ERP) in response to the last word of the sentence. The N400 is a useful tool to study expectation violation during language processing and is characterized by a negative deflection in the averaged electroencephalogram (EEG) peaking around 400 ms after a semantic violation (Kaan, 2007; Kutas and Federmeier, 2011). The amplitude is increased (i.e., more negative) for words that do not match the previously given semantic context and are hence unexpected (e.g., “*The pizza is too hot to cry*.”, Hillyard and Kutas, 1983). A small number of studies have investigated the N400 mimicking differential aspects of conversations, such as, for example, the effect of long or short pauses after questions in conversations (Bögels et al., 2015b). However, whether the N400 effect, the difference between expected and unexpected items, is a useful means for studying prediction building during conversation is not well studied.

Our design allows to study the effect of a semantic manipulation *during* a turn take within a sentence context on the N400, while still providing sufficient control to allow for a quantitative analysis (e.g., predefined sentences and precise timing information of the final word). Speech utterances in one of our conditions (reading aloud) are usually related to large muscle artifacts in the EEG, masking the signal of interest. We are able to circumvent this problem in this paradigm by analyzing only the sentence final word, i.e., the word that is generated by another speaker, providing us with an artifact free data segment.

Central to this paradigm is the semantic violation for the sentence's final word during the switch from one speaker to another (i.e., an element of turn-taking). For this we expected to observe the N400 effect for incongruent sentence endings compared to congruent endings. We expected an N400 effect despite speaker-switch and independent of the participant's previous role (speaking vs. listening) as in both cases one has to process how well the last word fits the formed expectation.

## Materials and methods

Sixteen healthy German-native speakers (eight female, mean age 24.1 years) took part in this study. All were right-handed according to the Edinburgh Handedness Inventory (Oldfield, 1971). Participants gave written informed consent prior testing and were paid for participation. The study was approved by the Ethics Committee of the University of Oldenburg and conducted in accordance with the declaration of Helsinki.

**Figure 1:**
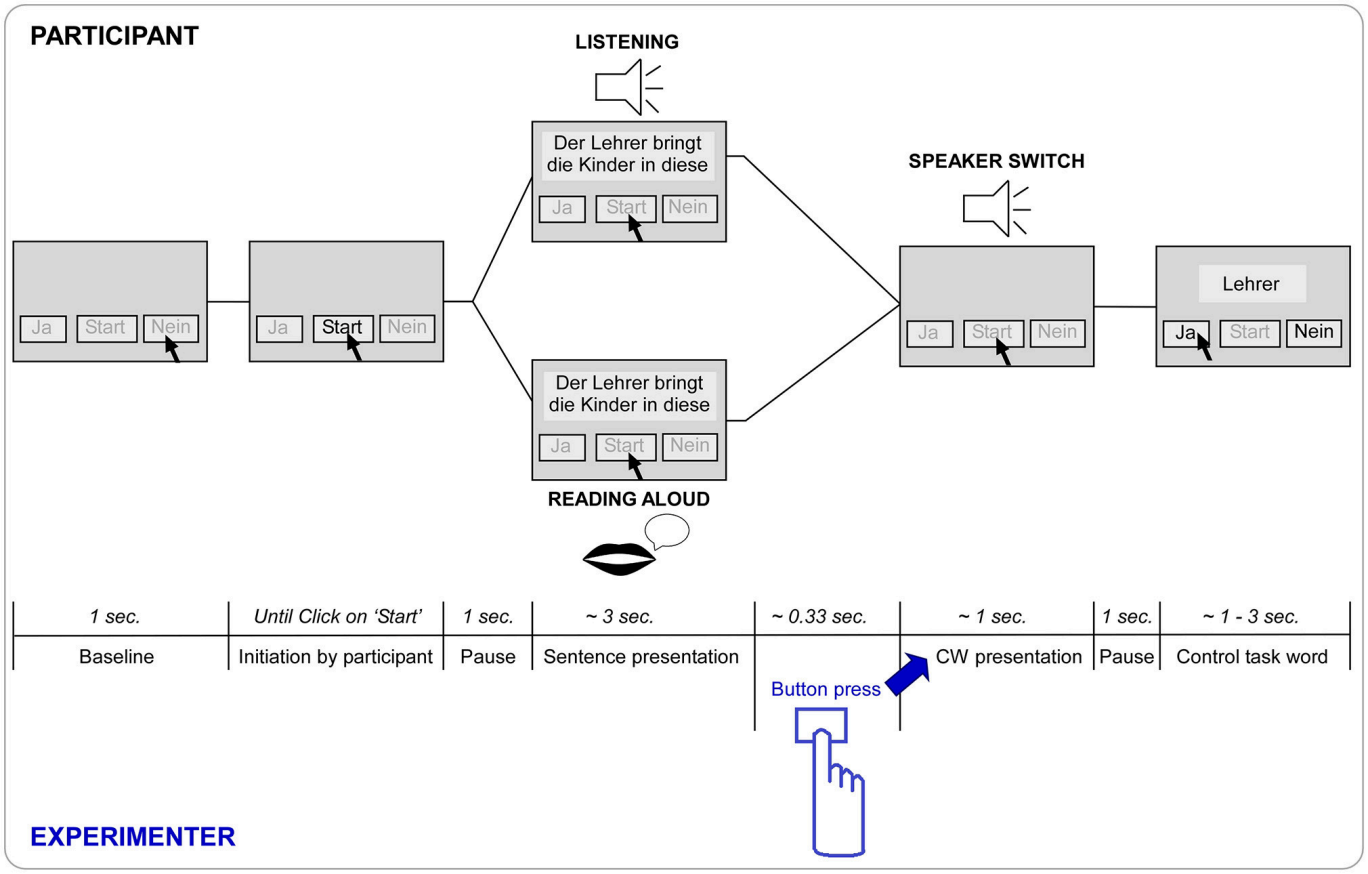
**Paradigm trial timeline in seconds and respective screen state**. After a baseline of 1 s the “Start” button is enabled and the trial is initiated by a click of the participant. After a pause of 1 s the respective first seven words of the sentence are presented simultaneously on the screen. Dependent on block the participant listens to a recording of the first speaker saying the first part of the sentence (Listening) or reads the first part of the sentence out aloud on its own (Reading aloud). The sentence is then completed by the second recorded speaker (speaker switch) saying the final (eighth) word of the sentence (= critical word CW), triggered by the button press of the experimenter (see highlights in blue). After a pause of 1 s the control task word appears on the screen and the “yes” (“Ja”) and “no” (“Nein”) buttons are enabled. As soon as the participant has clicked or maximum after 3 s, the word disappears. After a baseline of 1 s the next trial can be initiated again.

EEG was recorded from the participants in two turn-taking modes: (1) “Listening,” the mode of listening to the pre-recorded sentence fragment, which was also visible on the screen, and (2) “Reading aloud,” the mode of reading aloud the sentence fragment presented on the screen (Figure 1). To keep the two conditions as similar as possible, the sentence fragments were presented visually in both modes. The last word of the sentence (from now on critical word or CW) was, in both conditions, presented auditorily by another speaker and either completed the sentence in an expected semantically congruent or an unexpected semantically incongruent way. This resulted in a 2 (Listening vs. Reading aloud) × 2 (Congruent vs. Incongruent) design with the four conditions Listening congruent, Listening incongruent, Reading aloud congruent, and Reading aloud incongruent.

To control for the participants task involvement, a control word was presented after each sentence. Participants had to indicate whether this word was present or absent in the sentence by either pressing the left or the right screen button, respectively.

### Linguistic material

The linguistic material was adapted from the study of Ruigendijk et al. (2015). The sentences were balanced for word frequency and word length and a plausibility judgment for congruent and incongruent sentences was performed by native speakers. In brief, the material consists of 40 German eight-word sentences with *subject–predicate–direct object–goal/direction* pattern (e.g., “*The teacher takes the kids into this school*,” from German: “*Der Lehrer bringt die Kinder in diese schule*”). For each of these sentences a set of four sentences was generated, where the verb and the goal/direction (i.e., last word) was constant, while the subjects and direct objects where changed but still semantically closely related (e.g., “*The father takes the sons into this school*,” “*The mother takes the daughters into this school*,” and “*The bus driver takes the pupils into this school*”). This resulted in 160 semantically congruent sentences. Semantically incongruent sentences were created by replacing the final word with another final word (out of the 40 final words used, e.g., “*The teacher takes the kids into this wound*”). In this study we used in total a set of 80 incongruent sentences, providing us 40 incongruent sentences for the listening condition and 40 incongruent sentences for the reading aloud condition. We created two lists, each containing 160 sentences in total: 120 semantically congruent (with three presentations of the same CW) and 40 semantically incongruent sentences (i.e., the incongruent sentences were non-repetitive for each participant, whereas there was some repetition of congruent sentences). The order of the sentences was pseudo-randomized in five different versions for each list, to avoid order effects but to control for sequential presentation of semantically incongruent endings (≤5). Eight further unique sentences and unique final words were used for a practice block only.

The auditory material used for the sentences in the listening condition and the critical words for both conditions were recorded by two female speakers. The complete sentences were all recorded by one speaker (instructed to read in natural sentence intonation) and later cut, resulting in an auditory file with the first seven words of the sentence (mean sentence fragment length: 2.69 s, range 1.88–3.98 s). The final (eighth) word closing the sentence was recorded by the second speaker (mean word length: 0.71 s, range 0.46–0.98 s). To improve natural sentence intonation for the final words, an additional person was present during the recordings reading out the first part of the sentence. All auditory stimuli (44.1 kHz, 16 bit, WAV format) were edited with Praat (Boersma, 2001). A cosine ramp with a duration of 5 ms was implemented at the end of the seven-word sentences to remove any clicks after cutting and to smoothen the transition to the final word (see Auditory Material in the Supplementary Material for examples). All stimuli—sentences and final words—were adjusted to the same volume. Voice onset times for the final words were defined within millisecond range and set as stimulus onset time for analysis.

### EEG recording

Brain electrical activity was recorded with a wireless EEG system (http://www.mbraintrain.com). The EEG is recorded from 24 sintered Ag/AgCl electrodes (international 10/20: Fp1, Fp2, F7, Fz, F8, FC1, FC2, C3, Cz, C4, T7, T8, TP9, TP10, CP5, CP1, CPz, CP2, CP6, P3, Pz, P4, O1, and O2, reference: FCz, ground: AFz) with a small wireless amplifier (Smarting mBrainTrain, Belgrade, Serbia) attached to the back of the cap (Easycap, Herrsching, Germany). Recordings were digitized (Smarting Software 2.0.0, Smarting mBrainTrain, Belgrade, Serbia) with a sampling rate of 500 Hz and sent to a computer via Bluetooth. Electrode impedances were kept below 10 kΩ.

### Procedure

The EEG cap was set up and the individual participant was seated in the sound-attenuated testing cabin in front of the computer screen (≈60 cm). A short practice block was used to familiarize the participants with the task.

Each participant underwent two testing blocks—Listening and Reading aloud. The order of the testing blocks was randomized over participants. Two different stimuli lists were used for the first and second block (see Section “Linguistic Material”).

Each trial was initiated by the participant by clicking the “Start” button (Figure 1). The first seven words of the sentence were shown on the screen as text, and either presented also auditorily via loudspeakers (Listening) or read aloud by the participant (Reading aloud), which was followed by the auditory presentation of the critical word (CW, and speaker-switch) presented via loudspeakers. The final word was triggered by the experimenter with a button press in both modes (to distribute variance of gaps similarly). After the end of the critical word there was a pause of 1 s and the control word was presented on the screen (Figure 1). The participant was instructed to click “yes”/“no” indicating presence/absence of the control word in the sentence. In 50% of the trials the control word was present in the first part of the sentence and in the other 50% of the trials it was absent from the sentence. The control word was never the critical word. The task was implemented to make sure the participant was paying attention to the stimuli, as indicated by correctly categorized words (Duncan et al., 2009). In case of no answer, the word disappeared after 3 s. Participants were instructed not to swallow or move and to blink as little as possible from the onset of each trial (click on “Start”) until completion of the control word task.

An analysis of the gaps between sentence fragment and critical word was performed to ensure that no systematic differences in gap length were present between the modes. The average gap for Listening was 0.339 s long (*SD* = 0.019, range 0.163–1.048 s) and for Reading aloud 0.322 s long (*SD* = 0.020, range 0.130–1.531 s). No significant differences in gap length between the two turn-taking modes were present [paired *t*-test, *t*_(15)_ = −1.83, *p* = 0.088].

The experiment was programmed in Matlab R2012a (Mathworks Inc., Natick, MA), using the psychophysics toolbox (Brainard, 1997; Pelli, 1997). A microphone in the testing cabin recorded the trials and enabled the experimenter to listen to the utterances of the participant in the testing cabin. Lab Streaming Layer (https://github.com/sccn/labstreaminglayer, Lab Recorder, version 1.05) was used to record and synchronize all data streams (EEG signal, EEG markers, and microphone signal).

### EEG analysis

All trials were incorporated in the analysis as the participants rated on average 99.26% words correct in the control task (≈158.81 out of 160 trials; average Listening congruent 99.11%, Listening incongruent 99.38%, Reading aloud congruent 99.53%, and Reading aloud incongruent 98.75%).

EEG analysis was performed with EEGLAB (version 13.4.4b, Delorme and Makeig, 2004).

For artifact attenuation with independent component analysis (ICA) data were high-pass filtered at 1 Hz and low-pass filtered at 60 Hz [Finite impulse response filter (FIR), window type “Hann,” cutoff frequency −6 dB]. Dummy epochs (1000 ms) unrelated to the task structure were generated and all epochs displaying two or more standard deviations from the mean signal were rejected. Extended infomax ICA was applied and unmixing ICA weights were saved with the raw data.

For ERP analysis the raw data with ICA weights was high-pass filtered at 0.1 Hz and low-pass filtered at 30 Hz (FIR filter, window type “Hann,” cutoff frequency −6 dB). Artifactual ICA components were identified by visual inspection and removed. Data was then re-referenced to the arithmetic mean of the electrodes that were located on the left and right mastoid (TP9, TP10), epoched around CW onset (−50 ms to +1000 ms), and baseline corrected (−50 to +50 ms). This peristimulus baseline (compare Blackford et al., 2012) was used to ensure that no prior influence of either turn-taking mode (i.e., speech output in the reading aloud mode or auditory input in the listening mode) was present in the baseline signal. Analysis of the gaps between sentence fragment and critical word (compare “Procedure”) confirmed prior offset of speech (Reading aloud) and recording (Listening) to the selected baseline time window (−50 ms to CW onset).

An automatic artifact rejection procedure was used to exclude trials. Epochs displaying three or more standard deviations from the mean signal were rejected. The mean number of trials entering statistical analysis for Listening congruent were 111, Listening incongruent 37, Reading aloud congruent 111, and Reading aloud incongruent 33.

### Statistical analysis

For the definition of an unbiased time window for analysis of the N400 effect, we created a grand average across all participants including all conditions (Listening congruent and incongruent, Reading aloud congruent and incongruent) and electrodes (compare Keil et al., 2014, p. 7). Three components were present in the ERP: a negative peak at 140 ms, a positive peak at 254 ms, and a negative peak at 446 ms. Based on this grand average (see Supplementary Figure 1 in Data Sheet 1) we defined a window of analysis relying on the zero crossings of the ERP: a window for analysis of the N400 from 370 to 530 ms and for the P200 from 166 to 336 ms, relative to CW onset. Both effects, N400 and P200, were analyzed using repeated measures analysis of variance (ANOVA).

For analysis of the N400 effect, we conducted separate repeated measures ANOVAs along the midline and quadrants to evaluate its topographical distribution (compare Kos et al., 2010). The midline analysis included the within-subjects factors electrode (Fz, Cz, CPz, Pz), turn-taking mode (Listening, Reading aloud), and congruency (congruent, incongruent). In case of a significant interaction between electrode and congruency, follow-up single ANOVAs for each electrode were computed. The quadrant analysis included four quadrants (averaging over four electrodes), with the within-subjects factors quadrant (left anterior: Fp1, F7, FC1, C3, left posterior: CP5, CP1, P3, O1, right anterior: Fp2, F8, FC2, C4, right posterior: CP6, CP2, P4, O2), and the main factors turn-taking mode (Listening, Reading aloud) and congruency (congruent, incongruent). In case of a significant interaction between quadrant and congruency, follow-up single ANOVAs for each quadrant were computed.

To address concerns of differing signal-to-noise ratios between congruent and incongruent conditions (~120–40 trials, respectively) the midline and quadrant analyses of the N400 effect were repeated with matching numbers of trials, i.e., 40 trials for the congruent condition and 40 trials for the incongruent condition (see Supplementary Material, Data Sheet 1).

The P200 component has a fronto-central distribution with no clear lateralization (cf. Lee et al., 2012; Schierholz et al., 2015). Therefore, the analysis of this component was limited to frontal electrodes. The selection of the specific frontal electrodes for analysis of the P200 was based on the grand average topography including all conditions (Listening congruent and incongruent, Reading aloud congruent and incongruent) which led to the electrodes Fz, FC1, FC2, and Cz. The P200 statistical analysis included the within-subjects factors electrode (Fz, FC1, FC2, Cz), turn-taking mode (Listening, Reading aloud), and congruency (congruent, incongruent). In case of a significant interaction between electrode and congruency or electrode and turn-taking mode, follow-up single ANOVAs for each electrode were computed.

For all analyses, whenever Mauchly's test indicated a violation of sphericity, the Greenhouse-Geisser corrected *p*-values with the original degrees of freedom will be reported. In all cases, we focused on the results of the main effects of congruency, turn-taking mode, and their interaction; as well as the possible interaction of these main effects with the factors electrode or quadrant. Estimates of the effect sizes are reported in terms of partial eta squared (η_*p*_^2^) values.

## Results

**Figure 2:**
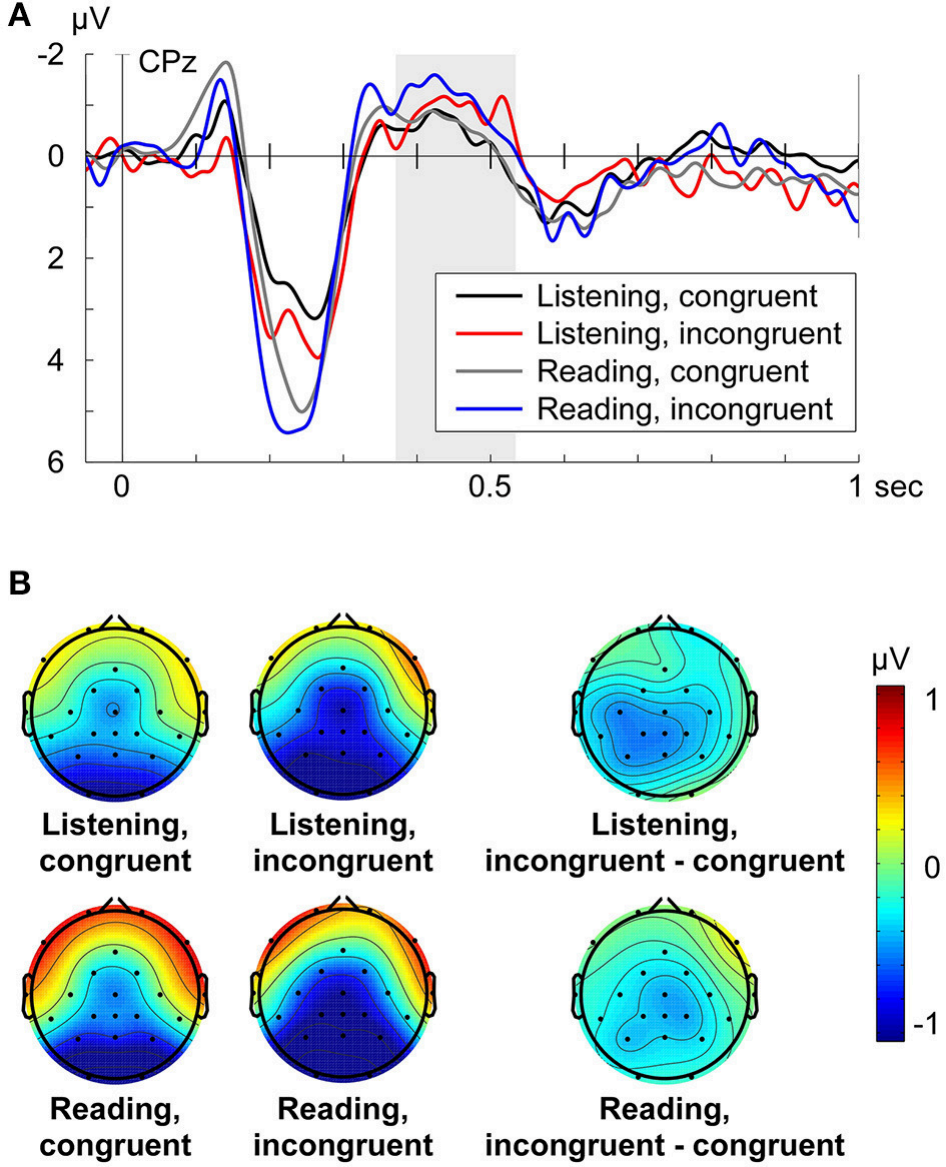
**(A)** Grand average ERPs at electrode CPz for each condition (Listening congruent: black, Listening incongruent: red, Reading aloud congruent: gray, Reading aloud incongruent: blue) respective to −50 to +50 ms baseline. Zero point is the onset of the critical word. **(B)** Grand average topographies of the N400 from 370 to 530 ms for each condition and the N400 effect (incongruent minus congruent condition) from 370 to 530 ms for each turn-taking mode. Electrode positions are displayed as black dots. Voltage scale is shown on the right.

The grand average ERP (e.g., CPz shown in Figure 2A) for Listening and Reading aloud was characterized by three dominant peaks: A negative deflection around 140 ms, a large positive deflection at around 200 ms (P200), and the negative deflection at around 400 ms, the time window in which the N400 effect can be expected to occur. For both turn-taking modes (Listening, Reading aloud) the amplitude of the N400 was larger for the incongruent condition compared to the congruent condition (Figures 2A,B).

### N400 effect

For analysis of the N400 effect, a separate midline (factor electrode: Fz, Cz, CPz, Pz) and lateral (factor quadrant) repeated measures ANOVA for the time window 370–530 ms was computed with the main common factors congruency (congruent vs. incongruent) and turn-taking mode (Listening vs. Reading aloud). The midline analysis of the N400 revealed that congruency (congruent vs. incongruent) had a statistically significant effect on the N400 amplitude [midline: *F*_(1, 15)_ = 6.07, *p* = 0.026, η_*p*_^2^ = 0.288], where incongruent conditions led to a larger N400 (Figure 2A). The turn-taking mode (Listening vs. Reading aloud) did not significantly affect the N400 amplitude [midline: *F*_(1, 15)_ = 0.11, *p* = 0.741] and there was no significant interaction between congruency and mode on the N400 amplitude [midline: *F*_(1, 15)_ = 0.26, *p* = 0.62]. We found a significant interaction between electrode and congruency [midline: *F*_(3, 45)_ = 4.54, *p* = 0.015, η_*p*_^2^ = 0.232] pointing to a distributional difference of the N400 effect along the midline (Figure 2B). Follow-up analyses revealed a significant N400 effect (i.e., a main effect of congruency) at electrodes Cz [*F*_(1, 15)_ = 5.13, *p* = 0.039, η_*p*_^2^ = 0.255], Cpz [*F*_(1, 15)_ = 7.63, *p* = 0.015, η_*p*_^2^ = 0.337], and Pz [*F*_(1, 15)_ = 6.82, *p* = 0.02, η_*p*_^2^ = 0.313]. To check whether these N400 effects were similar across turn-taking modes (Listening, Reading aloud) a follow-up Pearson's linear correlation analysis of the mean N400 effect (i.e., incongruent minus congruent mean ERP amplitude) across electrodes Cz, Cpz, and Pz was computed. It showed a significant correlation of the N400 effect (*r* = 0.43, *p* = 0.049) across turn-taking modes.

The analysis of the N400 among quadrants (left anterior: Fp1, F7, FC1, C3, left posterior: CP5, CP1, P3, O1, right anterior: Fp2, F8, FC2, C4, right posterior: CP6, CP2, P4, O2) also displayed a significant main effect of congruency on the N400 amplitude [quadrant: *F*_(1, 15)_ = 5.45, *p* = 0.034, η_*p*_^2^ = 0.266]. The turn-taking mode did not significantly affect the N400 [quadrant: *F*_(1, 15)_ = 0.04, *p* = 0.844] and there was no significant interaction between congruency and turn-taking mode [quadrant: *F*_(1, 15)_ = 0.93, *p* = 0.35]. We did not find a significant interaction between quadrant and congruency [quadrant: *F*_(3, 45)_ = 1.329, *p* = 0.28] suggesting a widespread N400 effect distribution. The results of the same trial number analysis were in line with the present results (see Supplementary Material, Data Sheet 1).

Visual inspection of the N400 effect topographies (Listening and Reading aloud: incongruent minus congruent, Figure 2B) suggested a slightly left-lateralized and parietal distribution. The grand average topography of the incongruent condition showed a more central and posterior distribution of the N400 within the analyzed time window (Figure 2B: Listening, incongruent and Reading, incongruent). This slightly posterior gradient of the N400 effect was confirmed by the midline analysis (compare above). These findings are in line with the reported distributions of the N400 component (cf. Hagoort and Brown, 2000; Kos et al., 2010; Wang et al., 2012; for a review see Kutas and Federmeier, 2011).

### P200

**Figure 3:**
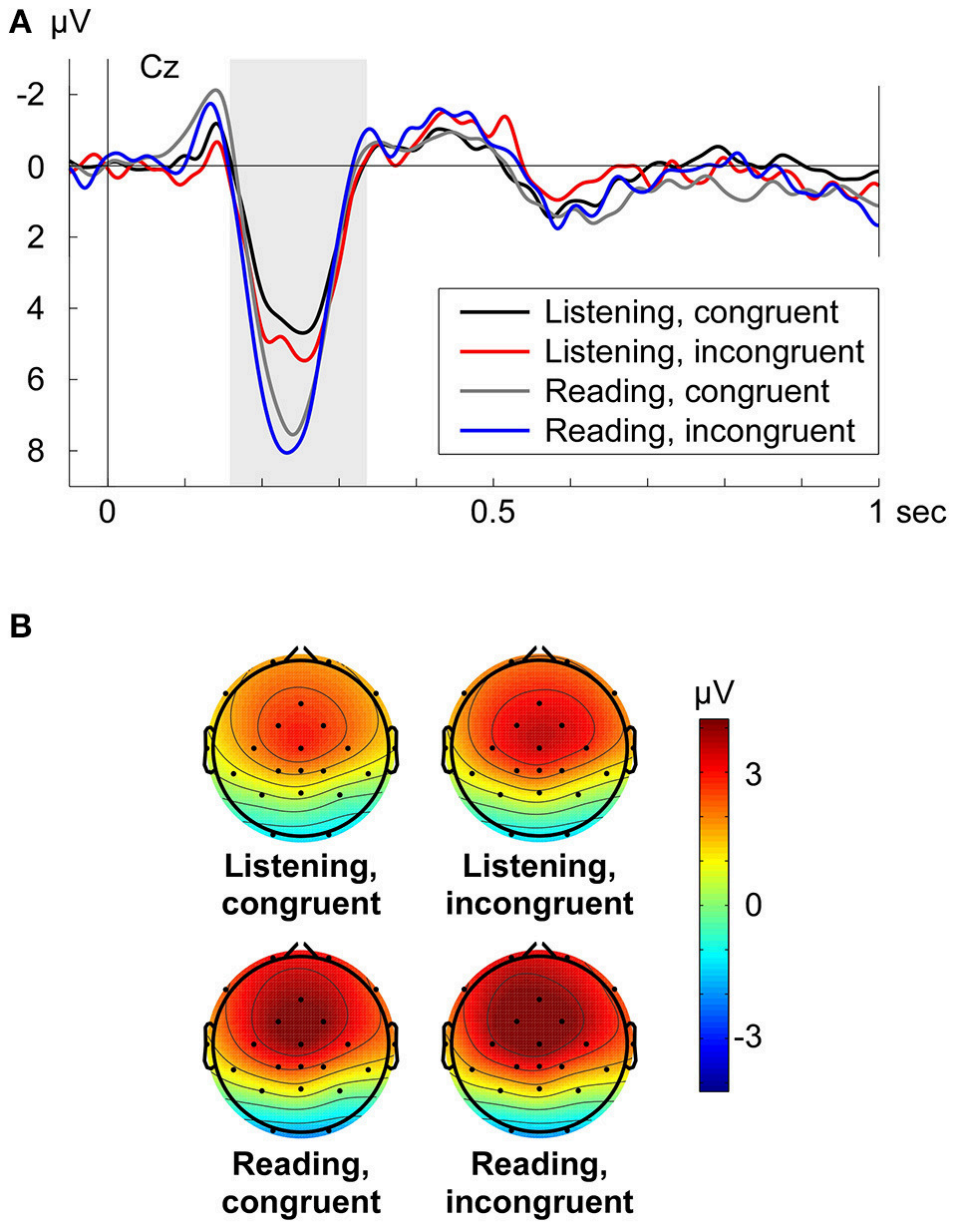
**(A)** Grand average ERPs at electrode Cz for each condition (Listening congruent: black, Listening incongruent: red, Reading aloud congruent: gray, Reading aloud incongruent: blue) respective to −50 to +50 ms baseline. Zero point is the onset of the critical word. **(B)** Grand average topographies for the P200 from 166 to 336 ms for each condition. Electrode positions are displayed as black dots. Voltage scale is shown on the right.

The P200 (Cz shown in Figure 3A) was analyzed with a repeated measures ANOVA in the time window from 166 to 336 ms along frontal electrodes (factor electrode: Fz, FC1, FC2, Cz) with the main factors congruency (congruent vs. incongruent) and turn-taking mode (Listening vs. Reading aloud). Congruency was marginally significant on the P200 amplitude [*F*_(1, 15)_ = 4.56, *p* = 0.05, η_*p*_^2^ = 0.233], where incongruent conditions showed an enhanced positivity (Figure 3A). In contrast to the N400 effect, the turn-taking mode (Listening/Reading aloud) had a significant effect on the P200 amplitude [*F*_(1, 15)_ = 7.71, *p* = 0.014, η_*p*_^2^ = 0.340], visible in higher amplitudes for the Reading aloud mode compared to the Listening mode (Figure 3A). There was no significant interaction between congruency and mode on the P200 [*F*_(1, 15)_ = 0.38, *p* = 0.548]. Further, no significant interactions between electrode and congruency [*F*_(3, 45)_ = 1.33, *p* = 0.276] or between electrode and turn-taking mode [*F*_(3, 45)_ = 1.64, *p* = 0.214] were present.

The grand average topographies for the analyzed P200 time window displayed the same fronto-central distribution over all four conditions (Figure 3B). In line with the statistical analysis, it was also visible that the P200 was increased in the Reading aloud mode compared to the Listening mode. Incongruent conditions too, had a slightly increased P200 in contrast with the congruent conditions (Figure 3B). Grand average difference topographies of the P200 can be found in Supplementary Figure 2 (Supplementary Material, Data Sheet 1).

## Discussion

Our research aim is to move toward neurophysiological studies of natural dialogues. The present study is a first step with regard to loosening some of the classical constraints that are posed on experimental paradigms by the recording setup. We investigated the N400 effect in a controlled turn-taking scenario using wireless EEG. Our results show that the expectancy violation in response to incongruent words is measurable as an amplitude increase in the N400 compared to congruent words. This was the case for both measured scenarios: when reading aloud the first sentence fragment and listening to the final word, as well as when listening to the first sentence fragment and the final word from different speakers. In other words, this introduced speaker-switch for the final (analyzed) word did not prevent detecting the N400 effect. The effect on the N400 was further present independent of whether the sentence fragment is uttered by oneself or by another person (i.e., reading aloud or listening). The topographies we found for the N400 effect are in line with earlier studies (Hagoort and Brown, 2000; Kos et al., 2010; for a review see Kutas and Federmeier, 2011; Van den Brink et al., 2012; Wang et al., 2012; Erlbeck et al., 2014). Unexpectedly, we found a large P200 component, which was also enhanced for the reading aloud condition compared to the listening condition. We ascribe this difference to the saliency of the new stimulus (auditory speaker-switch) and the differences between the modes in sound source and sound generation. In summary, this new paradigm has proven valuable in investigating the effects of a speaker-switch on the N400. The findings of this study provide a framework for future studies of neural correlates of language processing in more natural conversational setups, that inevitably incorporate turn-taking (and hence speaker-switches). A direct comparison of a speaker-switch condition and a non-speaker-switch condition might also be valuable for further research.

The amplitude difference between expected and unexpected sentence endings of the observed N400 effect within this turn-taking paradigm was moderate in comparison to other literature findings (Hagoort and Brown, 2000; Kos et al., 2010, but see also Gunter et al., 2000; Rueschemeyer et al., 2014). It is therefore possible that the participants did not process the sentences in depth. While the control task (word present or absent in sentence) ensured attention to the sequence of words, it did not ensure that the sentence was processed semantically (for a study of effects of task instructions on the N400 see Erlbeck et al., 2014). We expect that questions that require a more in-depth semantic analysis of the sentences (e.g., “Who took the children to the school?”) would lead to an increased level of engagement, in turn, this would drive expectations for the continuation of the sentences (cf. Van Berkum, 2012) which should also affect the N400 amplitude. Due to the design of the language material (see Section Linguistic Material), the N400 effect could also have been influenced by variations in the expectedness of specific sentence endings (i.e., the cloze probability for the specific critical words). A further reason for the rather small N400 effect could also be that the unexpected sentence endings of our study are not only unexpected but anomalous. As a result, participants might have stopped semantic analysis in some cases, leading to no enhanced integration costs and again to a less pronounced overall N400 effect (cf. Kutas and Federmeier, 2011).

Apparent in the data is the strong P200 for both turn-taking conditions. In general, the P200 (and N100, auditory evoked potentials) have been linked to higher order processes of attention and perception (Beauducel et al., 2000; Federmeier et al., 2005; Heim and Alter, 2006) and respond strongly to salient sound stimuli (i.e., novel or differing in intensity). We expect that this attention response is reflected in our P200 findings, that is, a strong orientation toward the new auditory stimulus: the speaker-switch. Further, we find an enhanced P200 for the reading aloud condition in contrast to the listening condition. Several factors might play a role here. First, in the reading aloud condition the participant has to switch from an active (i.e., reading aloud the first part of the sentence) to a passive task (i.e., listening to the critical word), while in the listening condition this switch is absent. Second, in the reading aloud condition there is a change in the sound source. On the one hand, the origin of the sound changes from the mouth of the participant to the loudspeaker, and on the other hand, the sound changes from self-generated to externally generated. Taken together, the sound properties in the reading aloud condition between the first part of the sentence and the last word differ more dramatically than in the listening condition. As the difference between the sentence and the critical word is more pronounced in the reading aloud condition, the higher amplitudes in this condition can be ascribed to that.

Our focus in this study was on the effect of a speaker switch and a semantic manipulation on the N400. Note, however, that we found congruency to be marginally significant on the P200 (*p* = 0.05), such that we cannot exclude an influence of congruency on the differences of the P200 amplitude. Previous studies presenting written material have reported similar results of an enhanced early positivity for semantically unexpected sentence endings compared to expected ones, which seem to interact with several factors, such as word frequency and repetition effects (Lee et al., 2012; Wang et al., 2012). In the present study, the critical word is presented auditorily but preceded by written (Listening and reading aloud condition) and spoken (Reading aloud condition) material. The interaction of these factors and their impact on the P200 remains to be studied. The absent interaction of congruency and turn-taking mode on the P200 points to two possibly different processes of congruency and physical stimulus analysis that are reflected in the P200.

The overall goal of our research is to approach neural correlates of natural conversational situations. As a first step, we integrated a speaker-switch for each congruent or incongruent ending word of a sentence within this turn-taking paradigm. The participant read aloud the first part of the sentence or listened to the first part of the sentence, but the last word was always presented by another speaker. An advantage of this speaker-switch setup was the separation of overt speech and the word of interest. This way, artifacts arising from mouth movements during speech could be circumvented for the signal of interest. To ensure the transition between the sentence fragment and the final word in this reading aloud condition, a human involvement for triggering the final word was necessary. As a result, the presentation of the last word of each sentence was triggered by the experimenter; only after she pressed the button the final word was presented. Engaging the experimenter had the advantage that the participant was able to read in the own preferred pace. Further, artifactual motor components arising from button presses could be evaded in the participant's EEG signal of interest. A drawback of human involvement is certainly the variability in reaction times. However, this should not have any effect on the studied EEG components. The analysis of the time between the sentence fragment and the final word shows no significant difference in the gap length for the two conditions. In addition, variable gap lengths between sentence fragment and final word are advantageous in terms of ecological validity. The gaps between utterances also vary during natural verbal interaction (Levinson and Torreira, 2015). Similarly, natural conversations include scenarios where the own utterance is completed by another person or one listens to someone else being completed (Coates, 1990; Purver et al., 2009). Both of these scenarios were included in the present paradigm (listening mode and reading aloud mode). In conversations, finishing each other's sentences is usually attested in situations where speakers seem to have lost their track of argumentation (e.g., helping out with a specific word) or to emphasize specific aspects of a discussion (Purver et al., 2009; Levinson and Torreira, 2015). Here, we mimicked the first case of helping out with a word in an active (reading aloud) and passive (listening) mode. In both modes, we predicted that expectation violations (in our case semantic violations) are visible in an N400 effect. Our results support this hypothesis.

Evidently, the paradigm used here still lacks many aspects of natural conversations. Conversations are primarily characterized by two (or more) speakers taking consecutive full turns (i.e., a full sentence or number of sentences), one at a time, with no or almost no overlap (Levinson and Torreira, 2015). This aspect could not be addressed in the present setup.

Our study provides a promising starting point for future research. Future studies will have to increase the naturalness of conversational settings while keeping the constraints that are necessary to ensure quantitative analysis. These would include measuring two persons at the same time that interact with one another, moving from controlled language material to self-generated speech and natural (i.e., not pre-defined) turn-taking behavior. Some of these aspects already have been addressed separately (e.g., Jiang et al., 2012; Kawasaki et al., 2013; Himberg et al., 2015; Mandel et al., 2015; Torreira et al., 2015; Bögels et al., 2015a,b,c; Bašnáková et al., 2015) but it remains a challenge to combine these with neuroimaging. An advantage of a wireless (and hence mobile) EEG—as used here—is the possibility to measure electrophysiology outside the strongly controlled and restricted lab environment (e.g., Debener et al., 2012) and, moreover, in interactive situations of two or more participants in immediate proximity (meeting the described criteria for more naturalness described previously).

## Conclusion

The study of neural correlates of language in conversational settings is challenging. Here we have made an attempt to study the neurophysiological underpinnings of language in a controlled turn-taking scenario using wireless EEG. We have shown that we can measure the N400 effect reliably despite a speaker-switch. Future studies can build upon the approach presented here and move toward more conversational settings to study neural mechanisms of language processing.

## Ethics statement

The study was approved by the Ethics Committee of the University of Oldenburg. All participants signed written informed consent prior measurement.

## Author contributions

Study design: TG, MB, ER, SS. Conduction of study: TG, MB. Data analysis: TG, MB. Data interpretation: TG, MB, ER, SS. Manuscript writing: TG, MB, ER, SS.

## Funding

This research was funded by the Volkswagen Foundation (European platform) and funds from the Task Group 7 “BCI for Hearing Aids,” DFG Cluster of Excellence 1077 Hearing4all, Oldenburg, Germany.

### Conflict of interest statement

The authors declare that the research was conducted in the absence of any commercial or financial relationships that could be construed as a potential conflict of interest.
