# Corrigendum: The N400 Effect during Speaker-Switch—Towards a Conversational Approach of Measuring Neural Correlates of Language

**DOI:** 10.3389/fpsyg.2017.00998

**Published:** 2017-06-13

**Authors:** Tatiana Goregliad Fjaellingsdal, Esther Ruigendijk, Stefan Scherbaum, Martin G. Bleichner

**Affiliations:** ^1^Department of Psychology, European Medical School, University of OldenburgOldenburg, Germany; ^2^Cluster of Excellence Hearing4all, University of OldenburgOldenburg, Germany; ^3^Department of Dutch, University of OldenburgOldenburg, Germany; ^4^Department of Psychology, Technische Universität DresdenDresden, Germany

**Keywords:** mobile EEG, prediction, N400, social interaction, conversation, language, dialogue, turn-taking

In the original article, there was a mistake in Figure 2 **(B)** as published. The two right-sided difference topography visualizations (upper and lower) of the grand average of the N400 effect (incongruent minus congruent condition) for the two turn-taking modes were exchanged. The shown N400 effect plot for Listening is the N400 effect plot for Reading aloud, whereas the N400 effect plot shown for Reading aloud is the N400 effect plot for Listening. This only affects the visualization of the two difference topographies. The corrected Figure 2 appears below. The authors apologize for this error and state that this does not change the scientific conclusions of the article in any way.

**Figure 2:**
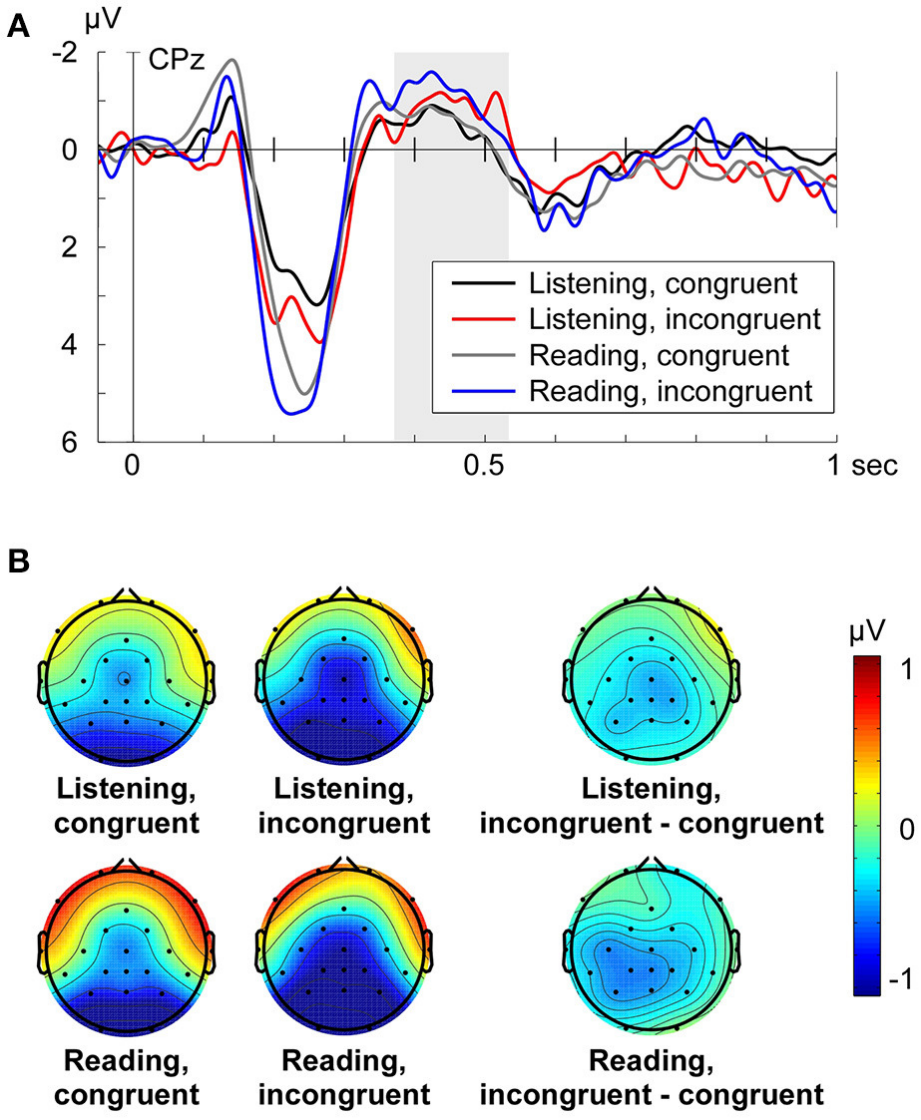
**(A)** Grand average ERPs at electrode CPz for each condition (Listening congruent: black, Listening incongruent: red, Reading aloud congruent: gray, Reading aloud incongruent: blue) respective to −50 to +50 ms baseline. Zero point is the onset of the critical word. **(B)** Grand average topographies of the N400 from 370 to 530 ms for each condition and the N400 effect (incongruent minus congruent condition) from 370 to 530 ms for each turn-taking mode. Electrode positions are displayed as black dots. Voltage scale is shown on the right.

## Conflict of interest statement

The authors declare that the research was conducted in the absence of any commercial or financial relationships that could be construed as a potential conflict of interest.

