# Supplementary material for: The N400 Effect during Speaker-Switch—Towards a Conversational Approach of Measuring Neural Correlates of Language
Source: Front Psychol. 2016 Nov 28;7:1854. doi: 10.3389/fpsyg.2016.01854 (PMC5124707; doi:10.3389/fpsyg.2016.01854)
Supplement: Supplementary file 1 [file DataSheet1.PDF]

Supplementary Material

The N400 effect during speaker-switch – Towards a conversational approach of measuring neural correlates of language

Tatiana Goregliad Fjaellingsdal*, Esther Ruigendijk, Stefan Scherbaum, Martin G. Bleichner

*** Correspondence:**
Tatiana Goregliad Fjaellingsdal
tatiana.goregliad@uni-oldenburg.de
Carl von Ossietzky Universität Oldenburg
26111 Oldenburg

1 Auditory Material

Twelve sentences (three sets with four sentences each), as used in this study, are presented exemplarily. One sentence out of each set has an incongruent final word and the remaining three sentences of the set have a congruent final word. The final word (critical word, CW) is highlighted in bold. An English translation of the sentence is given below the German sentence. The name of the sentence (e.g., Audio 1) corresponds to the name of the auditory wav-file online. A gap of 335ms (average gap of both modes) was inserted between sentence fragment and CW to simulate the auditory input during the Listening mode of the experiment.

- Audio 1 (congruent): ‘*Der Kapitän steuert das Schiff in diesen* ***Hafen****.*‘
  English translation: ‘*The captain steers the ship into this* ***port****.*’
- Audio 2 (incongruent): ‘*Der Matrose steuert das Boot in diesen* ***Stall****.*‘
  English translation: ‘*The sailor steers the boat into this* ***stall****.*’
- Audio 3 (congruent): ‘*Der Lotse steuert den Dampfer in diesen* ***Hafen****.*‘
  English translation: ‘*The pilot steers the steamship into this* ***port****.*’
- Audio 4 (congruent): ‘*Der Steuermann steuert die Yacht in diesen* ***Hafen****.*‘
  English translation: ‘*The helmsman steers the yacht into this* ***port****.*’
- Audio 5 (congruent): ‘*Der Autofahrer lenkt den Wagen in diese* ***Straße****.*‘
  English translation: ‘*The driver steers the car into this* ***street****.*’
- Audio 6 (incongruent): ‘*Der Busfahrer lenkt den Bus in diese* ***Flasche****.*‘
  English translation: ‘*The bus driver steers the bus into this* ***bottle****.*’

- Audio 7 (congruent): '*Der Taxifahrer lenkt das Taxi in diese* ***Straße****.'*
  English translation: '*The taxi driver steers the taxi into this* ***street****.'*
- Audio 8 (congruent): '*Der Chauffeur lenkt die Limousine in diese* ***Straße****.'*
  English translation: '*The chauffeur steers the limousine into this* ***street****.'*
- Audio 9 (congruent): '*Der Vater bringt die Söhne in diese* ***Schule****.'*
  English translation: '*The father takes the sons into this* ***school****.'*
- Audio 10 (congruent): '*Die Mutter bringt die Töchter in diese* ***Schule****.'*
  English translation: '*The mother takes the daughters into this* ***school****.'*
- Audio 11 (incongruent): '*Der Lehrer bringt die Kinder in diese* ***Wunde****.'*
  English translation: '*The teacher takes the kids into this* ***wound****. '*
- Audio 12 (congruent): '*Der Busfahrer bringt die Schüler in diese* ***Schule****.'*
  English translation: '*The bus driver takes the pupils into this* ***school****.'*

2 Average ERP

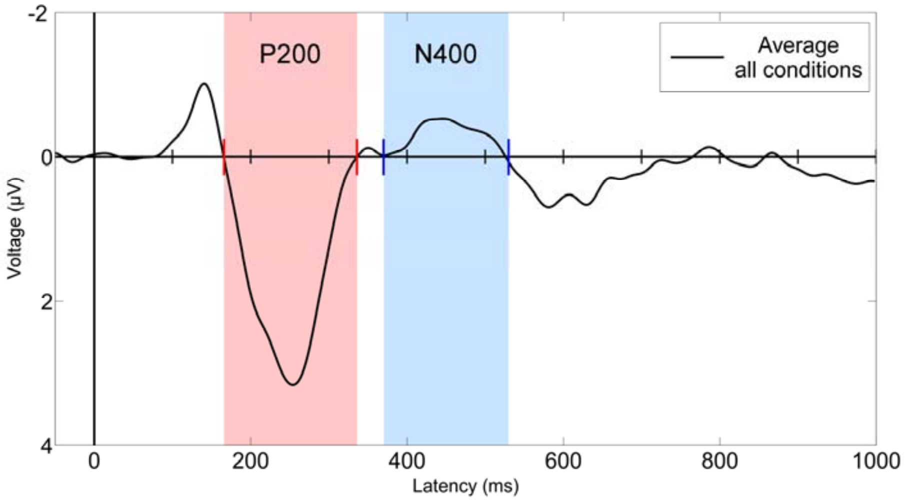

Supplementary Figure 1 Average ERP over all conditions (Listening congruent, Listening incongruent, Reading aloud congruent, Reading aloud incongruent), all subjects (n=16), and all electrodes (Fp1, Fp2, F7, Fz, F8, FC1, FC2, C3, Cz, C4, T7, T8, TP9, TP10, CP5, CP1, CPz, CP2, CP6, P3, Pz, P4, O1, and O2). The zero point is the onset of the CW. Negativity is plotted upwards. This average was used to define the time windows for statistical analysis of the ERP components. The P200 time window (166-336ms) is shown in red and the N400 time window (370-530ms) is depicted in blue.

3 P200 difference topographies

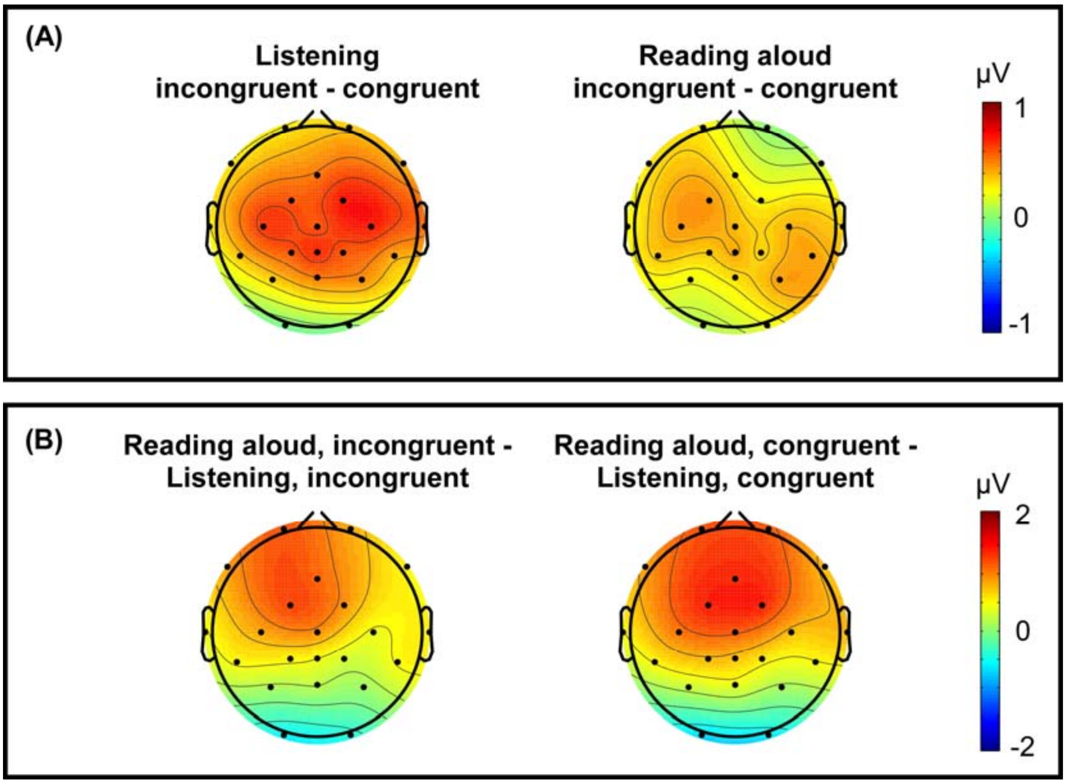

Supplementary Figure 2 Grand average difference topographies of the P200 from 166-336ms. (A) Left side: Listening incongruent minus Listening congruent. Right side: Reading aloud incongruent minus Reading aloud congruent. (B) Left side: Reading aloud incongruent minus Listening incongruent. Right side: Reading aloud congruent minus Listening congruent. Electrode positions are displayed as black dots. Voltage scale is shown on the right.

4 Same trial number analysis (N400 effect)

Signal-to-noise ratios may be different between congruent and incongruent conditions due to the differing trial numbers (~120 to ~40, respectively). This might affect the statistical analysis reported in the manuscript. To verify our results we repeated the analysis with matching trial numbers of congruent and incongruent conditions, i.e., 40 to 40 trials. We randomly selected for each critical word (40 in total) one out of the three possible trials of the congruent condition. Identical to the analysis in the manuscript, two separate repeated measures ANOVAs were computed, one along the midline [within-subject factors: electrode (Fz, Cz, CPz, Pz), turn-taking mode (Listening, Reading aloud), and congruency (congruent, incongruent)] and one along quadrants [within-subject factors: quadrants (left anterior: Fp1, F7, FC1, C3, left posterior: CP5, CP1, P3, O1, right anterior: Fp2, F8, FC2, C4, right posterior: CP6, CP2, P4, O2), turn-taking mode

(Listening, Reading aloud), and congruency (congruent, incongruent)] with the mean amplitude from 370-530ms.

The results of this analysis are in line with the results in the manuscript. The results showed that congruency had a significant effect on the N400 amplitude in the same trial number midline analysis ($F(1,15) = 8.98$, $p = .009$, $\eta_p^2 = 0.374$) and the same trial number quadrant analysis ($F(1,15) = 6.61$, $p = .021$, $\eta_p^2 = 0.306$), where incongruent conditions led to a more negative N400 amplitude. An interaction between electrode and congruency was present in the same trial number midline analysis ($F(3,45) = 4.89$, $p = .023$, Greenhouse-Geisser corrected). An interaction of quadrant and congruency was absent in the same trial number quadrant analysis ($F(3,45) = 1.18$, $p = .324$, Greenhouse-Geisser corrected). Turn-taking mode did not significantly affect the N400 amplitude in either analysis (same trial number midline: $F(1,15) = 0.16$, $p = .697$; same trial number quadrant: $F(1,15) = 0$, $p = .991$).

In summary, the same trial number analysis showed the same pattern as the analysis presented in the manuscript. From these results we conclude that the presented results in the manuscript are valid.
